# The evaluation of a slim perimodiolar electrode: surgical technique in relation to intracochlear position and cochlear implant outcomes

**DOI:** 10.1007/s00405-019-05696-y

**Published:** 2019-10-24

**Authors:** Floris Heutink, Berit M. Verbist, Lucas H. M. Mens, Wendy J. Huinck, Emmanuel A. M. Mylanus

**Affiliations:** 1 Department of Otorhinolaryngology, Radboudumc, Route 377, P.O. Box 9101, 6500 HB Nijmegen, The Netherlands; 2 Department of Radiology, Radboudumc, Nijmegen, The Netherlands; 3 Donders Institute for Brain, Cognition and Behaviour, Radboudumc, Route 780, P.O. Box 9101, 6500 HB Nijmegen, The Netherlands

**Keywords:** Cochlear implant, Electrode position, Imaging, Translocation, Residual hearing, Surgical approach

## Abstract

**Purpose:**

In cochlear implantation (CI), the two factors that are determined by the surgeon with a potential significant impact on the position of the electrode within the cochlea and the potential outcome, are the surgical technique and electrode type. The objective of this prospective study was to evaluate the position of the slim, perimodiolar electrode (SPE), and to study the influence of the SPE position on CI outcome.

**Methods:**

Twenty-three consecutively implanted, adult SPE candidates were included in this prospective cohort study conducted between December 2016 and April 2019. Mean age at surgery was 59.5 years. Mean preoperative residual hearing was 92.2 dB. Intra-operative fluoroscopy and high-resolution computed tomography scans were performed to evaluate electrode position after insertion using a cochleostomy (CS) approach. Follow-up was 12 months after implantation; residual hearing (6–8 weeks) and speech perception (6–8 weeks and 12 months) were evaluated in relation to the intracochlear SPE position.

**Results:**

In most patients in whom the SPE was positioned in the **s**cala tympani residual hearing was preserved [mean absolute increase in PTA of 4.4 dB and 77.2% relative hearing preservation (RHP%)]. Translocation into the scala vestibuli occurred in 36% of the insertions, resulting in a mean absolute increase in PTA of 17.9 dB, and a RHP% of 19.2%. Participants with a translocation had poorer speech perception scores at 12-month follow-up.

**Conclusion:**

Given the incidence of CS-associated translocations with the SPE and the negative effect on outcome, it is advised to insert the SPE using the (extended) round window approach.

## Introduction

### Rationale

The cochlear implant (CI) electrode array is the fundamental component of the CI system, as it provides the interface to the auditory system of the patient. Current CI electrodes are designed as either “precurved” or “straight”. Precurved electrodes are designed to curl around the medial wall and to assume a midscalar or perimodiolar position close to the modiolus, while straight electrodes assume a more lateral position, following the lateral wall of the cochlea [1]. Several studies have shown that perimodiolar electrodes, compared to lateral wall electrodes, lead to lower stimulation thresholds and reduced spread of excitation; stimulating a more specific, tonotopic region of spiral ganglion cells [2–7]. On the other hand, conventional perimodiolar electrodes translocate to the scala vestibuli (SV) at a higher rate compared to lateral wall electrodes [1, 8, 9]. These translocations are shown to be traumatic and are associated with loss of residual hearing and poorer speech perception [8, 10, 11].

To reduce intracochlear trauma during insertion, the slim perimodiolar electrode (SPE) was introduced in 2016. This electrode is 60% thinner and more flexible than the previous generation perimodiolar electrode produced by the same manufacturer [12]. The SPE has been developed for hypo-traumatic insertion and preservation of residual hearing. The surgical approach to the cochlea for the SPE is described as feasible using three surgical approaches to the cochlea: round window (RW), extended round window (eRW) or cochleostomy (CS) [13]. However, in previous generation electrodes, the approach to the cochlea has been shown to influence electrode position and audiologic outcomes. It was observed that independent of the type of electrode used, full scala tympani (ST) position [8] and preservation of residual hearing [8, 10] is more likely using a RW or eRW approach, compared to the CS approach. These findings might contribute to the fact that to-date, eRW and RW approaches were used in 91.8% (236/257) of the participants in the five studies that investigated the SPE [11, 12, 14–16].

The scalar position of the SPE, visualized in high-resolution imaging, was evaluated in 96 out of these 257 participants [11, 12, 15, 16]. Out of these participants, 81 were implanted using the RW or eRW approach [11, 12, 15, 16]; in 6 participants, a translocation had occurred [15]. In the remaining, relatively low number of 15 patients in whom the CS approach was used, no translocation was observed [12]. There is a need for more studies investigating the SPE, specifically that study correlations between the surgical approach and the electrodes’ position in the cochlea.

This paper reports on a prospective study with the SPE implanted using the CS approach in 23 participants. In a previous temporal bone study [17], as well as in clinical papers [12, 15], it was observed that there is a potential risk of tip fold-over when implanting the SPE. The primary objective of this prospective study was to evaluate the position of the SPE after a CS approach to detect translocations and tip fold-overs. A rotational flat, cone-beam computed tomography (CT) scan was used for the intra-operative fluoroscopy and CT-scan images. The secondary objective of this study was to investigate preservation of residual hearing in relation to the position of the SPE.

## Methods

### Study design and population

In this prospective study, 23 consecutive patients were implanted with the slim, perimodiolar electrode (SPE; Nucleus CI532; Cochlear Ltd, Sydney, Australia) between December 2016 and February 2018. All included patients were indicated for cochlear implantation, based on the Dutch CI indication criteria. As a part of the CI indication procedure, existing hearing aid fitting was optimalized, including fitting new hearing aids if deemed necessary. A CI was indicated if results in terms of aided speech performance with hearing aids is insufficient. Patients with functional residual hearing were informed about the study and invited to participate. Both patients with early and late onset of hearing loss were included in this study. Early onset of hearing loss was defined as an onset of hearing loss within the first 5 years of life. Patients with an early onset of deafness—i.e. prelingual onset of deafness—were excluded from this study.

Demographic data, history of hearing and pre-operative audiologic measurements were collected pre-operatively. All surgeries were performed in a hybrid operating theater equipped with a high-resolution, rotational cone beam CT scan (MITeC, Radboudumc, Nijmegen, The Netherlands). Intra-operative fluoroscopy and high-resolution rotational CT scans were performed after insertion. Post-operative residual hearing thresholds were measured at 2-month follow-up and speech perception, with electric stimulation only, was measured at 2- and 12-month follow-up. Approval was obtained from the Institutional Medical Research Ethics Committee (NL57456.091.16) and participants signed informed consent before participating.

### Electrode and procedure details

The SPE is precurved with an active length of 14 mm and a diameter of 0.35 mm × 0.4 mm at the tip, and 0.45 mm × 0.5 mm at the base. It is designed to provide full ST position with all common surgical approaches, including RW, eRW, or CS [13]. The SPE is loaded in an external, flexible silicon sheath, shaped as a tube, with a length of 5 mm, and then inserted together with the sheath inside the cochlea using two forceps, until the sheath stopper reaches the CS or RW opening. After insertion of the sheath, the electrode array is further inserted through the sheath at slow speed until full insertion (standardized insertion time ≥ 120 s). After full insertion, the sheath is retracted and removed. Surgery was performed by one surgeon (EM), using the standard mastoidectomy and facial recess approach. As our clinic had been selected by the manufacturer as one of the “early users group”, it was mandatory to undergo training with the SPE. In this training, the approach was an anterior–inferior positioned CS of which the diameter is checked with a silicone seizer tool which is included in the sterile blister package. All participants received a single dose of 1.8 mg/kg intravenous methylprednisolone during surgery. After full insertion of the electrode, the CS site was sealed with fragments of periosteum and fibrin glue.

### Electrode position evaluation

The Artis Zeego system (Siemens Healthcare, Forchheim, Germany), a multi-axis system for interventional imaging with a flat-panel detector, was used for intra-operative 3D imaging. Immediately after insertion, the surgeon used fluoroscopy imaging to rule out the presence of a tip fold-over. Post-operatively, the position of the SPE was evaluated on the CT images by an experienced Head- and Neck Radiologist (BV). For each of the 22 electrode contacts, the location was determined as either an ST or SV position.

### Audiologic assessment

Pre- and post-operative (2 months) unaided pure-tone thresholds at 125 Hz, 250 Hz, 500 Hz, 1000 Hz and 2000 Hz were measured both of the CI ear and the contralateral (CL) ear using a headphone in a soundproof room according to standard audiometric procedures. If the air-conduction threshold of the CI ear was 45 dB or higher—i.e. worse (Table 1), audiometric masking of the CL ear was performed using the standard plateau method according to Hood [18]. If a participant did not respond to an auditory stimulus, the threshold for that specific frequency was set at the maximum stimulation level (MSL). The MSL for the collected frequencies were 90 dB, 105 dB, 110 dB, 120 dB and 120 dB, respectively. This was in accordance with the consensus paper by Skarzynski et al. [19] on reporting on hearing preservation (HP). The absolute pure tone average of low frequencies (PTALow) was defined as the average threshold over frequencies: 250 Hz, 500 Hz and 1000 Hz. If a participant did not respond to two or more frequencies used for the calculation of PTALow, the PTALow was defined as non-measurable hearing (NMH). Fourteen patients were implanted in the poorer hearing ear, one had equal hearing thresholds in both ears and eight were implanted in the best hearing ear. Three patients who were implanted in the better hearing ear had limited difference in thresholds between the ears pre-implantation and symmetric vestibular function. These patients chose their ear to be implanted. One patient received his implant in the (slightly) better hearing ear because of good vestibular function in the worst ear and lack of vestibular function in the implanted ear. In the four patients that were implanted in the better hearing ear, the better hearing ear was the only ear with potential to reach speech perception performance with CI. Pre-implantation this ear had functional residual hearing, whereas in the CL, there had been a lack of auditory input for a long period, and therefore, poor performance was expected. These patients were advised to be implanted in the one hearing ear (Table 1). The mean absolute difference between the PTALow of both ears was 9.7 dB [standard deviation (SD) 8]. The difference between the pre-operative PTALow (prePTALow) and the post-operative PTALow (postPTALow) for the CI ear was defined as the absolute loss of the residual hearing (PTALowDiff). We used the HP classification system of Skarzynski et al. [19] to calculate the relative hearing preservation (RHP%): ${\text{RHP}}\% = \left[ {1 - \frac{{({\text{PTALowDiff}})}}{{({\text{PTALowMax}} - {\text{prePTALow}})}} \times 100} \right]$. PTALowMax-defined as the average MSL over the frequencies 250 Hz, 500 Hz and 1000 Hz was 111.7 dB. Based on their RHP%, each participant was categorized into one of the three defined categories of HP: (1) “Minimal HP” defined as RHP% between 0 and 25% (2) “Partial HP” defined as RHP% greater than 25–75% (3) “Complete HP” defined as RHP% greater than 75%.

**Table 1:** Characteristics of participants

Participant^a,b^	Age/gender	Duration of HL (years)	Etiology	CI side	Translocation to SV^b^	Pure tone average of low frequencies (in dB)^e^			Relative hearing preservation^f^		Post-operative speech perception (CVC-phoneme scores)		
						prePTALow CL ear	prePTALow CI ear	PTALowDiff CI ear	RHP%	Cat. HP	Pre-operative	2 months^c^	12 months^d^
1	56/M	21	Hereditary	L	Y	23	52	31.7	47	P	0	0	65
2	65/M	20	Hereditary	L	Y	77	83	21.7	24	M	3	56	–
3	45/F	45	Unknown	L	Y	88	90	20.0	8	M	9	45	44
4	64/M	10	M. Meniere	L	Y	92	92	20.0	0	M	20	56	56
5	67/M	14	Unknown	L	Y	87	95	15.0	10	M	0	63	65
6	58/F	20	Meningitis	L	Y	77	98	11.7	13	M	0	55	–
7	50/M	49	Meningitis	L	Y	82	103	7.5	25	M	9	39	48
8	58/F	57	Meningitis	L	Y	107	NMH	NA	NA	NA	6	45	47
9	60/F	52	Unknown	R	Y	62	NMH	NA	NA	NA	0	75	82
10	66/M	11	Unknown	L	N	62	67	11.7	74	P	42	69	95
11	44/F	42	Unknown	L	N	93	88	–	NA	NA	0	–	15
12	64/M	64	Unknown	R	N	92	88	8.3	64	P	46	45	45
13	55/M	37	Unknown	R	N	70	92	6.7	67	P	0	51	–
14	73/M	73	Unknown	L	N	105	95	11.7	30	P	6	71	62
15	43/F	43	Unknown	R	N	93	97	1.7	89	C	40	61	70
16	57/M	57	Usher Synd	L	N	NMH	98	− 2.5	125	C	0	–	–
17	44/M	44	Unknown	R	N	100	98	0.0	100	C	0	12	65
18	85/F	55	Unknown	L	N	NMH	102	0.0	100	C	6	–	–
19	56/M	51	Hereditary	R	N	NMH	103	5.0	40	P	0	66	56
20	69/F	25	Unknown	R	N	90	103	8.33	0	M	15	89	98
21	49/F	49	Unknown	R	N	103	108	− 2.5	160	C	0	–	–
22	78/F	30	Hereditary	L	N	80	NMH	NA	NA	NA	0	81	86
23	63/M	11	Unknown	L	N	73	NMH	NA	NA	NA	0	72	74

^a^Participant numbers 1, 2, 4, 5, 6, 9, 10, 13, 18, 20, 22 and 23 were defined as having late onset of hearing loss and participants numbers 3, 7, 8, 11, 12, 14, 15, 16, 17, 19 and 21 were defined as having early onset of hearing loss

^b^Participant number 3 is the one participant with tip fold-over and translocation of the four most apical electrode contacts

^c^CVC-measurement was not conducted at three months for participants 16, 18 and 21; as there was no understanding or use of spoken words in everyday life

^d^CVC measurement was not conducted at 12 months for participants 16, 18 and 21; as there was no understanding or use of spoken words in everyday life, and for participants 2, 6 and 13; because these participants were lost to follow-up during the first year

^e^Non-measurable hearing (NMH) is defined as a participant without response at maximum stimulation level on two or more frequencies

^f^Relative hearing preservation (RHP) is defined by Skarzynski et al. using the following formula: RHP = 100 × (1 − $${\raise0.7ex\hbox{${{\text{PTALowDiff}}}$} \!\mathord{\left/ {\vphantom {{{\text{PTALowDiff}}} {({\text{PTALowMax}} - {\text{prePTALow}})}}}\right.\kern-\nulldelimiterspace} \!\lower0.7ex\hbox{${({\text{PTALowMax}} - {\text{prePTALow}})}$}}$$)

In our clinic, speech perception in quiet is routinely measured at 2 and 12 months after implantation. The standard Dutch speech perception test of the Dutch Society of Audiology, which consists of phonetically balanced monosyllabic consonant–vowel–consonant (CVC) word lists, was used [20]. The average of 3 CVC lists (99 phonemes in total) was calculated. The test was carried out at 65 dB SPL, in a quiet audiometric booth, using a loudspeaker that was placed in front of the participant.

### Statistical analysis

Individual absolute and relative residual hearing thresholds and electrode position of participants are presented in Table 1. The results are grouped according to scalar position. Average absolute residual hearing thresholds and average RHP% were reported per group and between-group comparisons were performed using Student *t* tests (IBM SPSS Statistics 25.0) with the significance level set at 0.05 (Table 2). Speech perception scores of participants are reported in Table 1. As this was not an objective of the present study, and due to the heterogeneity of the data, these scores were not statistically analyzed.

**Table 2:** Mean loss of residual hearing in dB in participants with and without translocation to the scala vestibuli

	No translocation (SD)	Translocation (SD)	*P* value of Student’s *t* test^e^
Number of participants^a,b,c^	11	6	
Pre-op residual hearing (prePTALow in dB)^d^	95.4 (11)	87.1 (19)	0.26
Difference score^e^ (PTAlowDiff)	4.4 (5)	17.9 (9)	0.001
Relative hearing preservation according to Skarzynski et al.^f^ (%)	77.2 (45)	19.7 (16)	0.01

^a^One participant showed a tip fold-over and a translocation of the four most apical electrodes and was not included in the analysis

^b^One participant was lost to follow-up before post-operative residual hearing measurement and was not included in the analysis

^c^Four participants; two in each group, had no measurable hearing (NMH) pre-operatively and were not included in the analysis

^d^PTALow is defined as average pure-tone threshold over frequencies 250, 500 and 1000 Hz

^e^PTALowDiff is defined as average difference between post- and pre-operative PTALow thresholds

^f^Relative hearing preservation (RHP) is defined by Skarzynski et al. [19] using the following formula: RHP = 100 × (1 − $${\raise0.7ex\hbox{${{\text{PTALowDiff}}}$} \!\mathord{\left/ {\vphantom {{{\text{PTALowDiff}}} {({\text{PTALowMax}} - {\text{prePTALow}})}}}\right.\kern-\nulldelimiterspace} \!\lower0.7ex\hbox{${({\text{PTALowMax}} - {\text{prePTALow}})}$}}$$)

## Results

### Demographics

Twenty-three consecutive participants were included in this study, 13 males and 10 females. Twelve participants had a late onset of hearing loss. The average age at implantation was 59.5 (SD 11.0; range 43–85) years old and the mean preoperative residual hearing was 92.2 dB.

### Tip fold-over

**Fig. 1:**
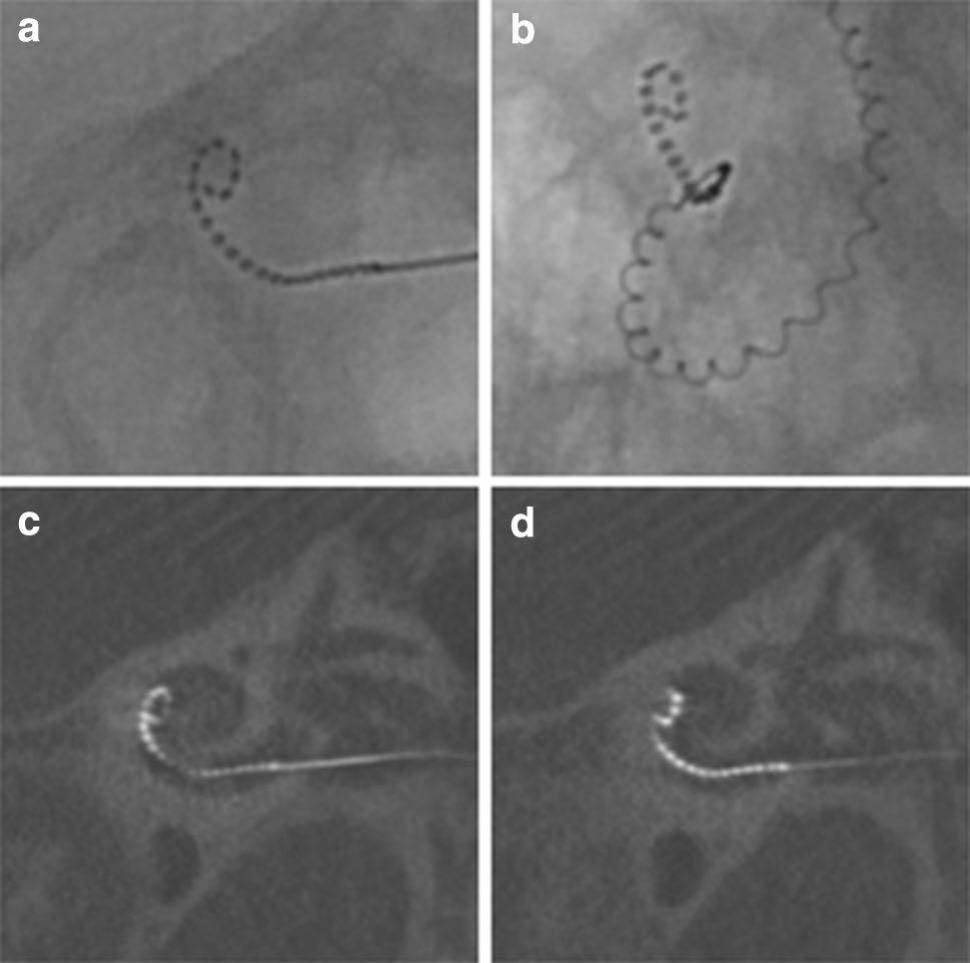
Images of the patient with a tip fold-over (**a**, **b** fluoroscopy, **c**, **d** CT-scan)

Intra-operatively, no tip fold-over was identified on fluoroscopy imaging. However, post-operative evaluation of the CT-images showed a tip fold-over of the 4 most apical electrode contacts in 1 of the 23 participants (4.3%). In retrospect, this tip fold-over was present on intra-operative fluoroscopy imaging, which was not recognized intra-operatively. Additionally, the SPE of this participant translocated from the ST into the SV at the location of this tip fold-over (Fig. 1). The participant, with early onset of hearing loss, decided not to be re-implanted; as speech perception was subjectively satisfactory and in-line with pre-implantation expectations. Loss of residual hearing (PTALowDiff) in this patient was 25 dB and CVC phoneme scores at 2 and 12 months post-operatively were 45% and 44%, respectively.

### Scalar position

**Fig. 2:**
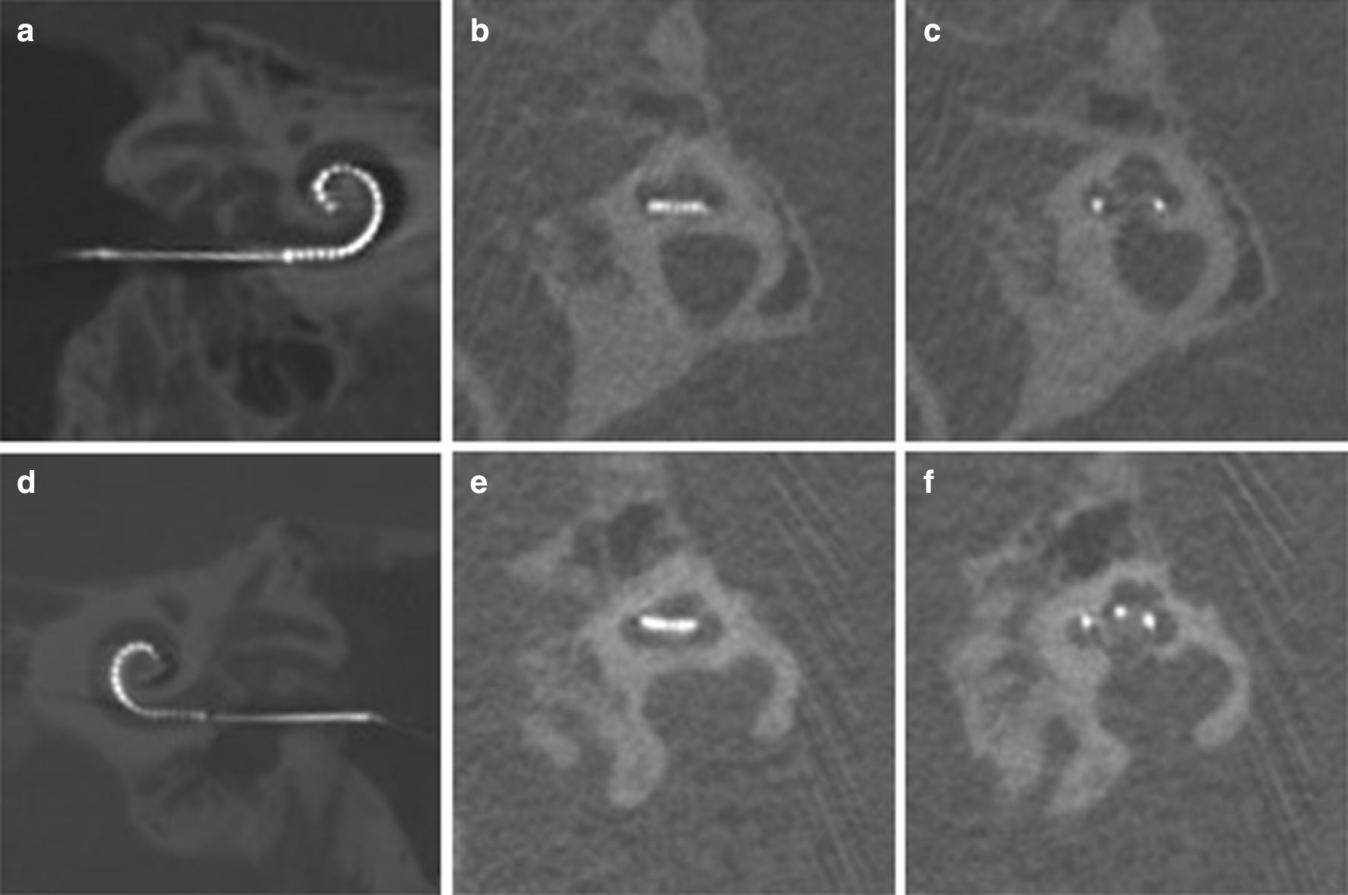
Position of different scalar locations (**a**–**c** scala tympani position, **d**–**f** scala vestibuli position)

In evaluation of the SPE scalar position of the 22 participants without tip fold-over, we found that 14 of the 22 participants (63.6%) had all electrode contacts positioned inside the ST. In eight participants (36.4%), the SPE translocated to the SV. All eight translocations occurred in near proximity of the CS. In 7 participants, all 22 electrode contacts were placed in the SV. In one participant, the first two contacts were located in the ST, before the SPE translocated to the SV. In Fig. 2, CT-scan images of an SPE in an ST position and in an SV position are shown.

### Audiometric outcomes

Table 1 shows the residual hearing thresholds for both ears and speech perception scores with CI for individual participants. The average loss of residual hearing was 9.8 dB (SD 9) across all participants with measurable pre-operative residual hearing and without a tip fold-over (*n* = 18). Table 2 shows an average absolute loss of residual hearing (PTALowDiff) of 17.9 dB (SD 9) in participants with translocation to the SV, while this was 4.4 dB (SD 5) in the participants without translocation (*p* = 0.001). Moreover, RHP in participants with and without translocation showed a similar statistically significant difference (*p* = 0.01); RHP% was 19.7% (SD 16) in patients with translocation and 77.2% (SD 45) when there was no translocation. With respect to the categorical relative HP, there was no participant of the translocation group (*n* = 7) with complete HP; one showed partial HP and six minimal HP. In the group of participants without translocation (*n* = 11), five showed complete HP, five had partial HP and in one patient, there was minimal HP.

The speech perception scores of participants with an early onset of hearing loss at 2 and 12 months post-implantation was lower (mean phoneme score, respectively, 48% and 50%) compared to the scores of participants with a late onset of hearing loss (mean phoneme score 61% and 78%, respectively). As shown in Table 1, overall, the individual speech perception scores at 2 and 12 months post-implantation of participants with complete ST position tend to be higher compared to scores of participants with translocation to the SV. No statistical analysis was performed on the differences in speech perception, as this was not an objective of the study, the population was too heterogeneous and the number of participants was too low to enable appropriate conclusions to be drawn.

## Discussion

The primary objective of this study was to evaluate the position of the SPE inside the cochlea after implantation using the CS approach. Intra-operative fluoroscopy and cone beam CT-scan were used for, evaluation of tip fold-over and scalar position, respectively. The secondary objective was to investigate the relationship between the position of the SPE and residual HP.

### Tip fold-over

One tip fold-over was found in this study (4.3%). In two other studies that investigated the SPE, 4.5% and 7.7% tip fold-over was reported [12, 15]. For comparison, in three studies, including conventional precurved CI electrode types, tip fold-over was found in 0.8%, 2% and 5.6% of the cases [21–23]. The slim and flexible design of the electrode is the obvious explanation for the higher frequency of tip fold-over in the SPE compared to conventional precurved electrodes. Ashendorff et al. [12] and McJunkin et al. [15] reported that, 88.9% (8/9) and 50% (1/2), respectively, of the tip fold-overs in the SPE were recognized in intra-operative imaging, and successfully re-inserted immediately. While this emphasizes the usefulness of intra-operative tip fold-over evaluation, it also demonstrates that recognizing a tip fold-over on fluoroscopy or plain X-Ray imaging may be challenging, especially if it concerns a limited number of apical electrode contacts. In the present study, the tip fold-over was missed during surgery. On the intra-operative fluoroscopy images, the tip fold-over had been misjudged as having a perimodiolar position (Fig. 1a, b). The tip fold-over became apparent once post-operative evaluation of the CT images clearly showed the relation to the cochlear wall (Fig. 1c, d). Surgeons implanting precurved electrodes, in particular the SPE, should perform intra-operative imaging, but should be aware of the challenges in evaluation the of fluoroscopy images.

### Scalar position

In 63.6% of the participants without a tip fold-over (*n* = 22), all electrode contacts were placed inside the ST, whereas 36.4% showed a translocation from the ST to the SV. In comparison, Shaul et al. [11], Aschendorff et al. [12] and Ramos et al. [16] found that 100% of in total 73 participants had full ST placement of the SPE; a CS approach was used in 15 participants, and the eRW or RW approach in 58 participants. While evaluating scalar location in 23/117 participants, McJunkin et al. [15] found 73.9% to have full ST insertion all implanted using the eRW approach. Interestingly, in this latter study, the participants with translocation to the SV had between 9 and 11 most basally located electrode contacts located in the ST; suggesting that the translocations occurred more apically compared to the translocations in our study. The fact that all translocations in our study were located directly at the CS site indicates a correlation with the used surgical technique. This hypothesis is strengthened by two studies [8, 24] with a large number of participants (*n* = 116 and *n* = 220); in which it was reported that a CS approach, compared to an (extended) RW approach, is associated with higher risk of translocation, independent of the implanted conventional electrode type. Our study is the first to report CS associated translocations for the slim, perimodiolar electrode, which was designed to be non-traumatic with any surgical approach [13].

Based on anatomical studies, it is advised that a CS should be located anterior–inferiorly or inferiorly to the RW to avoid direct translocation into the SV or direct damage to the basilar membrane [25–27]. The most straightforward explanation for the early translocations in the present study is that the CS was positioned too superiorly; resulting in the direct insertion in the SV, or in a scalar translocation, immediately after the electrode is inserted into ST. On the other hand, it seems unlikely that the position of the CS provides the full explanation. In this prospective study, following extensive training by the manufacturer in insertion of the SPE with positioning of an anterior–inferior CS, and meticulous use of the silicone gauge for size of the CS of 0.8 mm provided in the sterile implant package, the experienced surgeon was highly focused on a correct implementation of the CS position. A theoretical explanation, possibly relevant to the present study, is that a combination of factors, including the anterior–inferior CS, the size of the CS, the design of the insertion tool and flexible sheath, the angle of insertion, the force applied during insertion, and, in particular, the anatomical variation of the cochlea, play a role. Illustrative is an anatomical study of 73 cochleae in which the, for example, the height of the basal turn ranged from 1.6 to 2.6 mm (mean 2.1, SD 0.2 mm) [28]. Clinical relevance of anatomical variation in CI surgery was studied by Atturo et al. in 23 temporal bones [29]; the distances between the oval window, RW and spiral lamina were measured, and specifically compared in relation to CS sites located anterior–inferiorly, and inferiorly to the RW. The authors concluded that in a cochlea with small dimensions, only a very inferior CS could guarantee access to the ST without trauma to the spiral lamina. The obvious solution—to position the CS inferior to the RW—could be very challenging. Due to the fact that the SPE insertion is a two-hand procedure, the tool itself represents volume and the area inferior to the RW is difficult to access.

While the exact explanation for the (CS associated) translocations in present paper remains unclear, based on the findings of this study and the reports in the literature, in favor of the RW approach rather than the CS [8, 24], it was decided to convert our surgical approach for the SPE to the RW approach to ensure highest probability on the ST position. Extending the RW approach, which involves removal of the crista semilunaris and some of the anterior bony edge of the window with a diamond drill size 0.8 or even 0.6 mm, is necessary for the SPE to facilitate the insertion of the sheath of the insertion tool. Moreover, as in the present study, the CI surgeon is experienced and was specifically trained in inserting the SPE using the CS approach, it might be expected that other surgeons inserting the SPE using the CS approach also have a high risk on translocation. This emphasizes the importance of quality control with imaging.

### Audiologic outcomes

In this study, it was shown that the SPE can provide preservation of residual hearing (defined as RHL% > 75%), however, only if inserted non-traumatically. Participants with full ST position of the SPE array had an average loss of low frequency residual hearing (PTA3lowDiff) of 4.4 dB and RHP% of 77.2%. Yet, in participants with a translocation to the SV, we found a statistically significant higher average loss of low frequency residual hearing of 17.9 dB and low RHP of only 19.7%. The only other study that described translocations in the SPE [15] did not report on residual hearing thresholds specifically for the participants with translocation. The found average loss of PTA3lowdiff in participants without translocation (4.4 dB) was lower compared to the median loss of residual hearing (8.3.dB) found in an large multicenter study [30] that investigated the hybrid-L electrode; a short straight electrode that was specifically designed for preservation of residual hearing.

In this study, it was observed that participants with translocation to the SV had lower speech perception scores compared to participants with a ST position. These differences indicate that scalar position might not only be of importance for residual hearing, but also for speech perception results. While the finding that ST position is important for the best speech perception is in line with similar findings in literature [8, 10, 11, 31]; speech perception is influenced by several factors, which were not accounted for in this paper. Moreover, our study population was relatively small and heterogeneous due to variation in biographic and audiologic factors; e.g. etiology, duration of hearing loss, pre-operative speech perception and age at implantation. Authors of this paper would like to emphasize the findings on speech perception should, therefore, be interpreted with caution.

## Conclusion

In this prospective study, it was confirmed that the SPE carries a risk of tip fold-over—underscoring the need for intra-operative control. The slim perimodiolar electrode, once positioned in the ST can provide preservation of residual hearing. However, CS-associated translocation to the scala vestibuli occurred in more than one-third of the participants and was shown to be detrimental for residual hearing thresholds. Based on the results of the present study and evaluation of literature, if the anatomical situation allows it, we advise to insert the slim perimodiolar electrodes using the eRW approach.
